# Genetic variation in storage protein and starch biosynthesis genes reveals key associations with seed composition in pea (*Pisum sativum*)

**DOI:** 10.3389/fpls.2025.1679498

**Published:** 2025-12-05

**Authors:** Admas Alemu, Kavitha Anguluri, Bo Yuan, René Lametsch, Cecilia Hammenhag

**Affiliations:** 1 Department of Plant Breeding, Swedish University of Agricultural Sciences, Alnarp, Sweden; 2 Department of Food Science, Copenhagen University, Frederiksberg, Denmark

**Keywords:** *Pisum sativum*, storage proteins, starch pathway, genetic diversity, targeted gene sequencing, SNP markers

## Abstract

Understanding the genetic basis of seed storage proteins and starch biosynthesis in pea (*Pisum sativum*) is critical for breeding programs aimed at enhancing seed quality, functional properties and nutritional value. In this study, we selected 34 genes encoding storage proteins and 21 genes involved in starch synthesis through literature mining and BLAST analysis. Using these genes as references, we sequenced 64 genomic regions associated with storage proteins and starch metabolic enzymes in 100 genetically diverse global pea accessions. In addition, protein and starch content analysis was conducted for these pea accessions. Targeted sequencing of these genes yielded 8,793 genetic variants, of which 2,573 high-quality single nucleotide polymorphism (SNP) markers were retained for further analysis. Protein content of studied accessions ranged from 19.5 to 37.7%, and starch content from 23.8 to 47.3%, highlighting the substantial phenotypic variation. Furthermore, a strong negative correlation (r = –0.71, p < 0.001) was observed between seed wrinkling and starch content consistent with the documented role of genetic variation in starch biosynthesis in determining seed texture. The SNP-based population structure and genetic diversity assessments revealed a complex genetic landscape with no clear clustering by geographical origin or material types. However, dendrograms constructed from principal components derived from SNP markers revealed a clear clustering of accessions according to their protein and starch content, thereby validating the influence of variants in the targeted genes on these traits. Gene-to-phenotype regression analysis identified key genes significantly associated with protein and starch content including legumin, provicilin and starch-branching enzyme. These findings provide valuable insights into the genetic architecture of storage protein and starch biosynthesis in pea and offer a foundation for targeted breeding strategies aimed at improving seed composition and functional properties.

## Introduction

Pea, an annual cool-season crop belonging to the Fabaceae family, is the fourth most widely cultivated legume globally (Fraś et al., 2024). Its evolutionary origin traces back to the Mediterranean and Southwest Asian regions, where it was first domesticated approximately 10,000 years ago (Weeden, 2007; Zohary et al., 2012). Over millennia, domesticated pea landraces and cultivars have adapted to diverse climatic conditions, generating a vast genetic diversity globally that continues to support modern breeding programs (Rascio and Nicoletta, 2013).

Pea is an economically and nutritionally important crop with a significant role in agricultural systems world-wide (Parihar et al., 2023; Wu et al., 2023). With the increasing demand for plant-based protein sources, pea has become an increasingly important crop for both human nutrition and as a sustainable ingredient in animal feed (Tayeh et al., 2015; Parihar et al., 2023). In recent years, the demand for high-protein crops has increased due to efforts to transition toward more sustainable food systems, making pea an attractive target for breeding programs aimed at improving protein content. The objectives of such breeding programs are aligned with global nutrition security goals, addressing both consumer demand and the need for sustainable agricultural systems that support reduced greenhouse gas emissions and improved soil health (Wijerathna-Yapa and Pathirana, 2022).

The functional properties of pea ingredients, such as solubility and gel formation, play a pivotal role in their usability across various food products. Gel formation is influenced by the composition and structure of starch and storage proteins (Lu et al., 2020; Li, 2022). However, despite its importance, a complete understanding of the gel formation mechanisms of pea ingredients remains elusive, particularly in the context of broader genetic variation. These traits are crucial for developing next generation plant-based foods with desirable textures and consistency, making them competitive with conventional protein sources (Malalgoda et al., 2025; Sarathy et al., 2025). Storage proteins not only provide the primary source of nitrogen and essential amino acids but also influence the texture of pea-derived products (Grossmann, 2024). Similarly, starch composition, particularly the balance of amylose and amylopectin, and the proportions of their chain lengths, governs gelatinization temperatures and gel quality during processing (Ratnayake et al., 2002; Wang et al., 2024).

Most studies on functional properties of pea ingredients have been conducted using a limited genetic pool of modern cultivars, leaving the broader genetic diversity found in landraces and other more diverse plant materials largely unexplored (Carpenter et al., 2017). Despite the identification of key genes involved in storage protein synthesis and starch pathways, a comprehensive understanding of the genetic variation underlying these traits across diverse pea accessions remains limited. This gap is particularly relevant as breeding programs aim to optimize texture, stability and nutritional profiles of pea varieties. In this context, our study investigates the genetic polymorphisms in storage protein and starch-related genes using a diverse panel of 100 pea accessions comprising gene bank landraces, cultivars and breeding lines. By analyzing these polymorphisms, we aim to inform breeding strategies that enhance the functional and nutritional value of peas, ensuring their continued relevance in sustainable diets and agricultural systems.

## Materials and methods

### Plant material and DNA extraction

A diverse subset of 100 pea accessions was selected for this study to maximize genetic diversity leveraging insights from previous research by Brhane and Hammenhag (2024). Briefly, genetic distance was first estimated by grouping the 265 pea genotypes into six categories based on plant material type: wild, mutant, gene bank accessions (of largely unknown type), landraces, breeding lines, and cultivars. Principal Coordinate Analysis (PCoA) was then performed to visualize the genetic relationships among the genotypes. Based on the PCoA results, the genotypes were clustered into three distinct groups. Representative genotypes, positioned distantly within each cluster, were selected for the current study. This selection was further supported by Neighbor-Joining (NJ) clustering analysis. Detailed passport information and additional data for these accessions are provided in **Supplementary Table 1**.

Five seeds from each of the 100 accessions were separately sown in pots under a controlled greenhouse environment. After two weeks of growth, emerging leaves were sampled for genomic DNA extraction. For each accession, one leaf from a single plant was sampled into 2 ml tubes containing two 6-mm-diameter glass beads to facilitate subsequent homogenization. The leaf samples were lyophilized for 48 hours to ensure complete dehydration, which preserved DNA integrity and facilitated tissue disruption during homogenization. Dried tissue samples were then homogenized by vigorous shaking using a mixer mill (MM400, Retsch GmbH, Haan, Germany) to ensure thorough pulverization. Genomic DNA was extracted from the homogenized plant material using the GeneJET Plant Genomic DNA Purification Mini Kit (Thermo Fisher Scientific, Waltham, USA), following the manufacturer’s recommended protocol to achieve high yield and purity. DNA quality was initially assessed by running the extracted DNA on a 1% agarose gel electrophoresis, which allowed visual confirmation of DNA integrity. DNA concentration and purity were further measured using a Nanodrop spectrophotometer (DS-11 FX+, DeNovix Inc., Wilmington, USA) and the Quant-iT™ PicoGreen™ dsDNA Reagent and Kit (Thermo Fisher Scientific, Waltham, USA) to ensure precise quantification across samples. Finally, the samples were stored at -20 °C until they were sent for analysis.

### Identification of gene regions

A comprehensive literature review was performed to compile the genes of interest related to storage protein synthesis, starch metabolism and properties of gelatinization in peas and related legume species (Carpenter et al., 2017; Collins et al., 2021; Yu et al., 2021). When genes were identified in other species, their sequences were queried against the pea reference genome using BLAST. A maximum E-value threshold of 3e-69 threshold with more than 65.8% similarity was applied, representing the most permissive cut-off accepted for retaining significant matches. This approach led to the identification of 64 genomic regions in the pea genome associated with storage protein synthesis and starch metabolism. Among these regions, 34 genes were identified related to storage protein genes while 21 were corresponding to enzymes involved in starch metabolic pathways (**Supplementary Table 2**). To reduce sequencing costs, three larger genes (SBEI, ISA1, ISA3) were strategically divided and sequenced, focusing specifically on their exon regions. Flanking sequences were also included to differentiate closely related homologs. In total, 212,967 bp spanning the 64 gene regions, and flanking sequences across all seven chromosomes were sequenced.

### Targeted gene sequencing

The 64 identified genomic regions were sequenced using the 100 pea accessions following a targeted sequencing approach conducted by CD Genomics, (Shirley, USA). Approximately 500 ng of DNA/accession was taken for DNA sampling. High-quality genomic DNA was fragmented into sizes ranging from 220 to 450 bp using the Bioruptor Pico system (Diagenode, Belgium). The fragmented DNA was then repaired by treating with DNA-damage repair and A-tailing mix. Indexed adapters were ligated to both ends of the DNA fragments for sample identification following hybrid capturing using a custom-designed panel. Library quality was assessed using a Qubit fluorometer (Thermo Fisher Scientific, Waltham, USA) and a real-time PCR system, while the average fragment size was determined with an Agilent 2100 Bioanalyzer (Agilent Technologies, Santa Clara, USA).

The developed DNA libraries were then sequenced using the Illumina Novaseq or Hiseq PE150 platform (Illumina Inc., San Diego, USA). Paired-end sequencing was performed to generate high-quality reads with a target read length of 150 bp per read. Raw sequencing data were processed through a series of stringent quality control steps to generate clean data suitable for downstream analysis.

Several sequencing metrics were evaluated to ensure robust coverage and depth across target regions. SNPs and insertions/deletions (InDels) were identified using the Genome Analysis Toolkit (GATK) software and annotated in Variant Call Format (VCF) specification (v4.3).

The processed sequencing data were analyzed using custom scripts and bioinformatics pipelines to generate detailed coverage statistics, variant annotations and insights into genomic regions. Marker density was calculated for each gene and gene group (storage protein and starch pathway), followed by SnpEff analysis v.5.2c Ruden et al. (2012) where the sequenced regions are annotated against the pea reference genome (https://www.ncbi.nlm.nih.gov/datasets/genome/GCF_024323335.1/). The coding effects are taken into consideration based on their genomic location, excluding flanking regions for a detailed assessment of marker impact across different functional groups. Putative impact of high and moderate effects is considered to study the functional alterations in proteins and amino acid sequences. The different classes are defined as follow: “High-effect” variants include predicted stop-gained, frameshift, or splice-disrupting changes; “Moderate” include missense substitutions within coding regions; “Low/Modifier” cover synonymous or non-coding changes.

### Protein and starch content analysis

Protein and starch content analysis was performed on 96 of the 100 selected accessions comprising landraces, cultivars, breeding lines and genebank accessions with unknown pedigrees. The Dumas method was used to quantify nitrogen content in the pea flours using an Organic Elemental Analyzer (vario MACRO cube, Elementar, Hesse, Germany). Protein content of accessions was estimated from the nitrogen content analysis using a nitrogen conversion factor of 6.25. Although this factor can vary among legume species, reported values for peas range between 5.15 and 6.25 as it has been reported in literatures (Mariotti et al., 2008; Xing et al., 2025). Each sample was analyzed in triplicate.

Total starch content in the pea flour was measured using a Total Starch Assay Kit (Megazyme, Bray, Ireland) following AACC Method 76-13.01 as described by Dueholm et al. (2024). The resistant starch method (RTS-NaOH Procedure) was applied and each sample was measured in at least duplicates.

### Seed morphology phenotyping and correlation analysis

Seed wrinkling was assessed using the criteria described by the guidelines of the International Union for the Protection of New Varieties of Plants (Upov, 2009) and was recorded as a binary trait, classified as either wrinkled or smooth. Seed patterning was assessed visually as either uniform or patterned. For each accession, a minimum of 20 seeds was evaluated under consistent lighting conditions to ensure comparability. Seed size, expressed as profile area in square millimeters, was measured using a Marvin ProLine I seed analyzer (MARViTECH GmbH, Wittenburg, Germany). To assess the relationships among the protein, starch and seed shape parameters, Pearson’s correlation coefficients were calculated for continuous variables. Correlation between categorical and continuous variables was evaluated using one-way analysis of variance (ANOVA). For continuous traits and binary categorical variables, point-biserial correlation coefficients were calculated using Pearson’s method (Tate, 1954). For continuous traits and multi-level categorical variables, the strength of correlation was quantified using eta-squared (η²) from one-way ANOVA, with correlation-like coefficients computed as the square root of η² (Metsämuuronen, 2023). Correlations between categorical variables were analyzed using Chi-square test and the correlation coefficient was estimated using Cramér’s V coefficient (Cramér, 1999). All analyses were conducted in the packages *Hmisc* and *lsr* in the R environment v.4.3 (Team, 2023).

### Genetic diversity and population structure analysis

The selection of accessions for this study was informed by previous genetic diversity analysis by Brhane and Hammenhag (2024), which confirmed a wide genetic range suitable for evaluating population structure and trait associations. A neighbor-joining (NJ) analysis was conducted to cluster the 100 accessions using all identified SNP markers in the TASSEL software package v5 (Bradbury et al., 2007) and the resulting tree was visualized in the web-based program iTOL v 4.3.2 https://itol.embl.de/ (Letunic and Bork, 2019). All principal components were computed in TASSEL using the complete set of SNPs as well as subsets of SNPs specifically associated with storage protein and starch enzyme genes.

### Gene to phenotype regression analysis

A two-stage regression analysis was executed to evaluate the relationships between the SNP markers, the two phenotypic traits (i.e. protein and starch contents) and annotated genes.

In the first stage, the relationship between SNP markers and the measured phenotypic traits was evaluated using a general linear regression model using the *lme4* R package (Bates, 2016):


$$Y_{ij}\;=\mu \;+\beta G_{ij}\;+\;\gamma PC1_{i}+\;\gamma PC2_{i}+\epsilon_{ij}$$

Where: Y_ij_ is the phenotypic value (protein or starch) for the _i_th cultivar and _j_th SNP marker; μ is the intercept; β is the estimated effect of the _j_th SNP marker on the phenotypic trait; G_ij_ is the genotypic value of the _i_th accession at the _j_th SNP marker which was assigned as 0, 1 and 2 for homozygous major, heterozygous and homozygous minor alleles, respectively; γ is the coefficients for the two principal components (PC1 and PC2) to account for population structure; PC1 and PC2 are the values of the first two principal components derived from a principal component analysis (PCA) of the SNP markers; and ϵ_ij_ is the residual error assumed to follow a normal distribution with a mean of zero and variance *δ²* (ϵ_ij_ ∼ N(0, *δ²*)).

The model was fitted separately for each SNP, and markers showing a significant association with either trait were identified using a p-value threshold of 0.05. In the second stage, significant SNPs identified in the first stage were mapped to their annotated genes. For each gene, the weighted average effect was estimated providing greater influence to SNPs that had both stronger statistical significance and lower estimation uncertainty (Han and Eskin, 2012) using the following model:


$$E_{g}=\;\;\;\frac{\Sigma_{j\in s_{g}}w_{j\;}{\hat{\beta }}_{j}}{\Sigma_{j\in S_{g}}w_{j}}$$

Where $E_{g}\;$ is the weighted average effect of gene  g on the phenotypic trait; $S_{g}\;$ is the set of selected significant SNPs associated with gene  g; ${\hat{\text{β}}}_{j}$ denotes the effect of the _j_th SNP on the phenotypic traits estimated from stage one. $W_{j}$ is the weight assigned to SNP *j* which was calculated as the inverse of the squared standard error, $W_{j}=\;\frac{1}{SE_{j}^{2}}$ giving weights to SNPs with more precise effect estimates.

The weighted standard deviation ( ${\text{SD}}_{g})$ was also calculated for each gene following the method used by Savage et al. (2018) to quantify the dimension of variability among the effects of associated SNPs using the following model:


$${\text{SD}}_{g}=\sqrt{\frac{{\text{Σ}}_{\text{j}\in {\text{s}}_{g}}w_{\text{j}}({\hat{\beta }}_{j}\;-{\text{ E}}_{g})}{{\text{Σ}}_{\text{j}\in \text{s}g\;}w_{\text{j}}}}$$

Several packages found within *tidyverse* (Wickham et al., 2019) and other standalone packages such as *broom* (Robinson, 2014), *dendextend* (Galili, 2015)*, VCFR* (Knaus and Grünwald, 2017) and *ComplexHeatmap* (Gu, 2022) were employed for statistical modeling, manipulation, interpretation and to visualize the genotypic and phenotypic data in the R environment.

The dendrogram, constructed from the first two principal components (PCs) derived from SNP markers of the accessions was aligned with the phenotypic data for protein and starch content.

## Results

### Selection of candidate genes for sequencing and polymorphism screening

To explore genetic variation, our literature review in pea and related species identified 55 relevant genes comprising 34 for storage proteins and 21 for starch biosynthesis enzymes (**Supplementary Table 2**). These genes were selected based on their established or potential links to functional properties in peas, which directly influence texture and usability in food applications.

**Figure 1:**
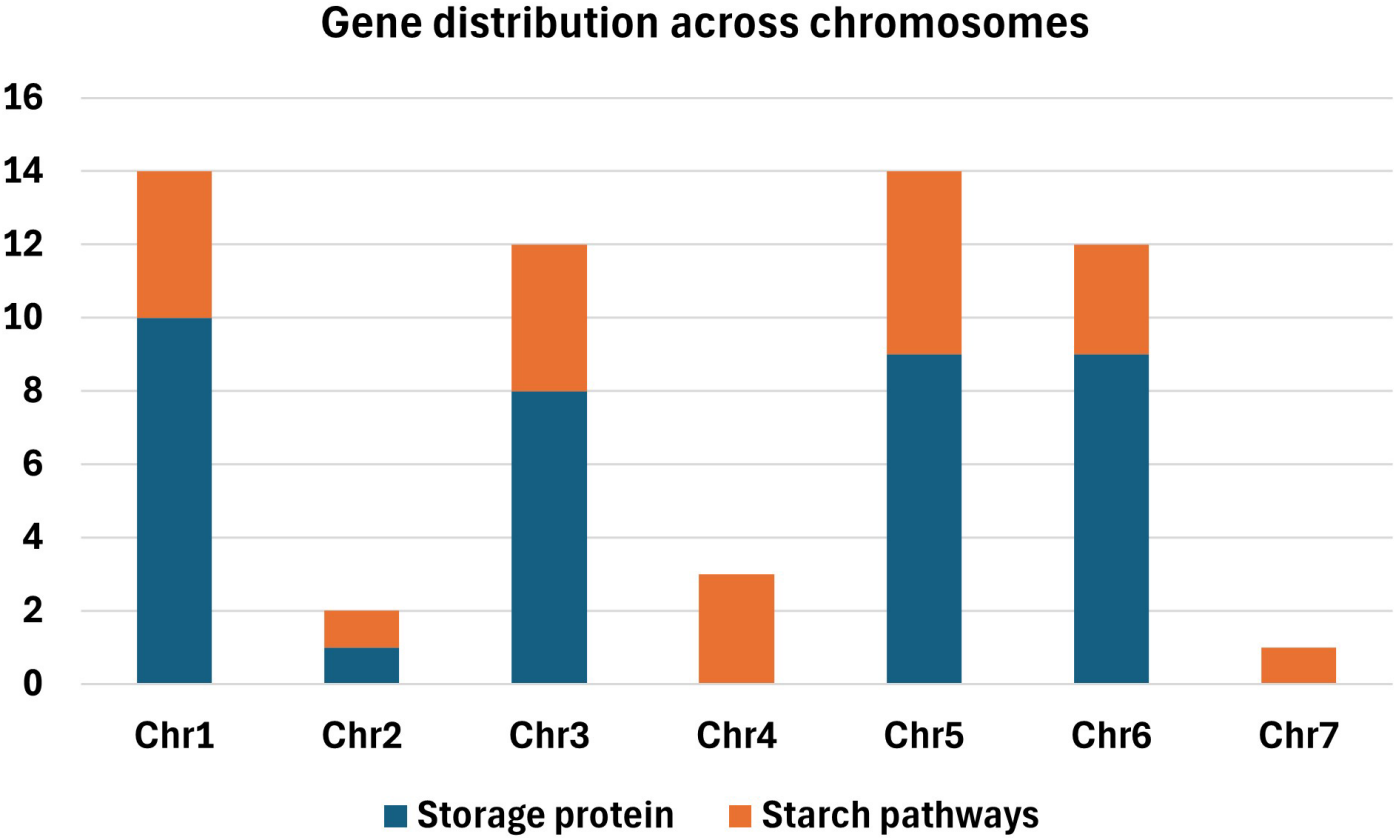
Distribution of 55 genes across the seven pea chromosomes (Chr), including 34 encoding storage proteins and 21 involved in the starch biosynthesis pathway. The dark blue bars represent storage protein genes, while orange bars denote starch pathway genes.

The distribution of the targeted genes across the pea genome varied across chromosomes, reflecting potential structural or functional specializations within the genome (**Figure 1**). Chromosomes 1 and 5 contained the highest number of genes with 14 genes identified on each. Chromosomes 3 and 6 also provided a substantial number of genes, with 12 genes each, whereas chromosome 4 contained three genes. Chromosome 2 contained a relatively low number of genes with only two, while chromosome 7 had only a single targeted gene.

The sequencing data from 100 accessions revealed a total of 8,793 genetic variants including 7,662 SNPs and 1131 insertion-deletions (InDels) across the seven pea chromosomes. The number of identified genetic variants covered all seven chromosomes with the highest on chromosome 5 while the lowest on chromosome 2. The downstream analysis including the genetic diversity, population structure and regression analysis with protein and starch contents was performed using SNP markers with a minor allele frequency (MAF) greater than 5% and with less than 20% missing data per accession. A total of 2,573 high-quality SNPs passed the filtering thresholds and were distributed across all seven chromosomes (**Table 1**).

**Table 1:** Distribution of genetic variants and SNP markers passing quality control thresholds across the seven pea chromosomes.

Chromosomes	Total genetic variants	Number of selected SNPs
1	1728	522
2	205	126
3	1614	474
4	300	163
5	2672	823
6	1144	393
7	230	135

**Figure 2:**
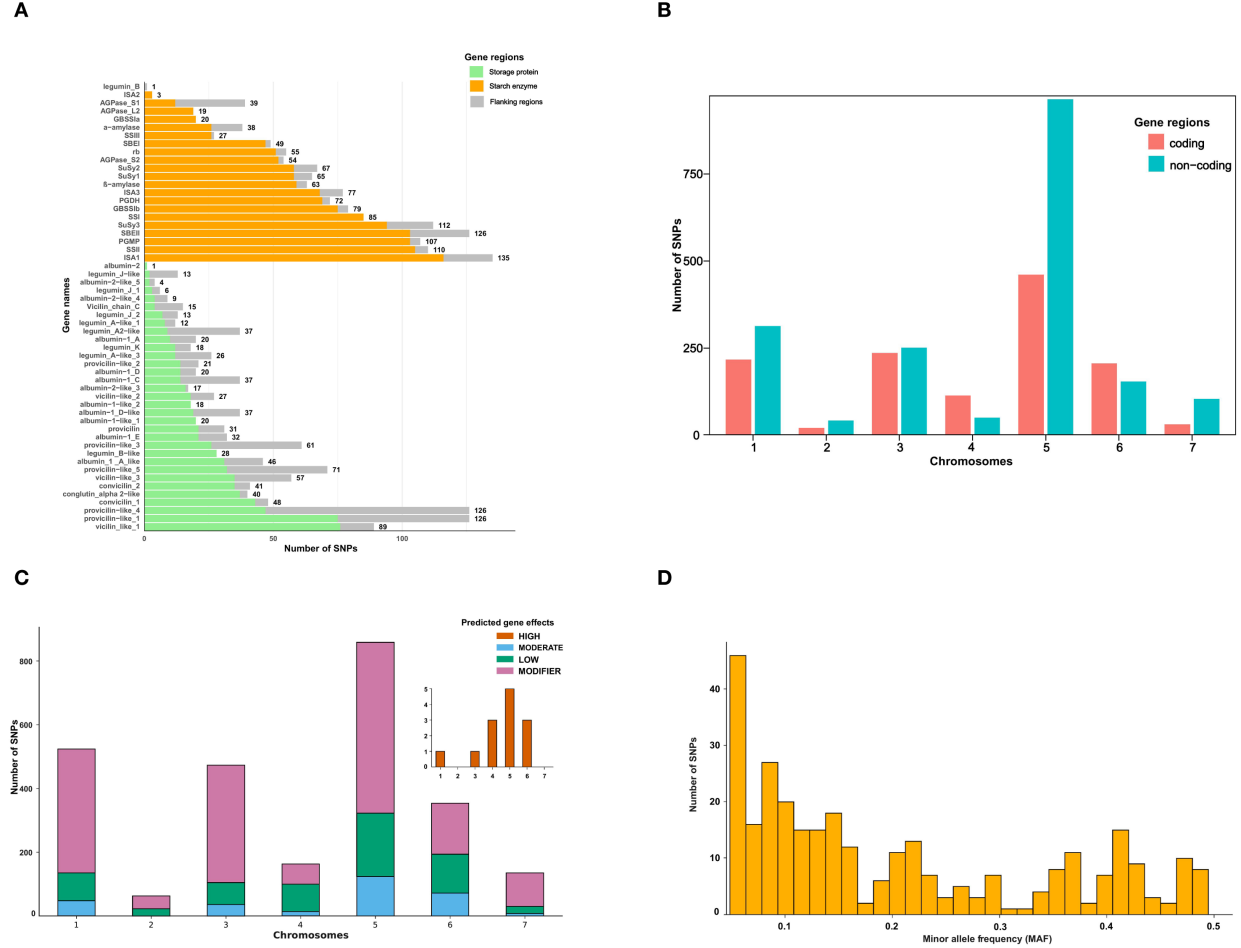
Distribution and characterization of single nucleotide polymorphisms (SNPs) identified across the targeted genes and genomic regions in *Pisum sativum*. **(A)** The number of SNPs identified per gene, categorized by genomic region as storage protein genes, starch enzyme genes, and flanking regions. Gene names are displayed on the y-axis, while the x-axis represents the number of SNPs detected per gene. **(B)** Distribution of SNPs across chromosomes, separated into coding and non-coding regions. The x-axis represents chromosome numbers (1–7), while the y-axis represents the number of SNPs detected in each region. Color of the plots represents the number of identified SNPs from either coding or non-coding regions. **(C)** Classification of SNPs based on their predicted functional impact. SNPs were categorized into four effect groups: high effect (brown), low effect (green), moderate effect (blue), and modifier (purple). The x-axis represents chromosome numbers (1–7), while the y-axis shows the number of SNPs per category. **(D)** Distribution of SNPs with moderate and high predicted effects based on minor allele frequency (MAF). The histogram shows the number of SNPs across MAF intervals, with a notable skew toward rare alleles (MAF < 0.1). The x-axis shows the distribution of SNPs according to their MAF values, while the y-axis shows the number of SNPs markers.

The sequenced target genes covered both the coding and non-coding regions associated with storage protein, starch enzyme and their flanking regions (**Figure 2**). Starch enzyme genes comprised a notably higher number of SNPs (1,249) compared to storage protein genes (714). The remaining 610 SNPs were identified from the flanking regions of targeted genes.

High quality SNPs were identified across the 55 genes with varied number of markers across these genes. Several genes encoding enzymes in the starch pathway such as ISA1, SuSy3, SSII, PGMP, and SBEII contributed to a higher number of SNP markers. The ISA1 carried the highest number of SNPs with 135, followed by SuSy3 and SSII contributing with 112 and 110, respectively. In contrast, albumin-2 and legumin_B, both encoding storage proteins, each carried only a single SNP marker (**Figure 2A**).

The highest number of SNPs from non-coding regions was recorded on chromosome 5 while the lowest on chromosome 2 (**Figure 2B**). Non-coding SNPs were more abundant than SNPs in coding regions on all chromosomes except chromosomes 4 and 6. Overall, chromosome 5 contained the most SNPs (863), followed by chromosomes 1 (522) and 3 (473).

SNPs were classified by predicted functional impact using *SnpEff*. Most SNPs were classified as modifier variants, followed by low-effect variants (**Figure 2C**). A total of 13 SNPs distributed on chromosomes 1, 3, 4, 5 and 6 were predicted to have a high effect. Another 296 SNPs were classified as having moderate effects. MAF values of the moderate and high-effect SNPs ranged from 0.05 to 0.495 across the 100 accessions. Of these, 98 had MAF below 0.1, while 81 SNPs had MAF exceeding 0.3 (**Figure 2D**). Of the 13 high-effect and 296 moderate-effect SNPs, several occur within conserved domains of storage proteins (vicilin/convicilin/legumin, conglutin-α2-like) and starch enzymes (SSII, SBEII, GBSSI).

### Genetic diversity analysis

**Figure 3:**
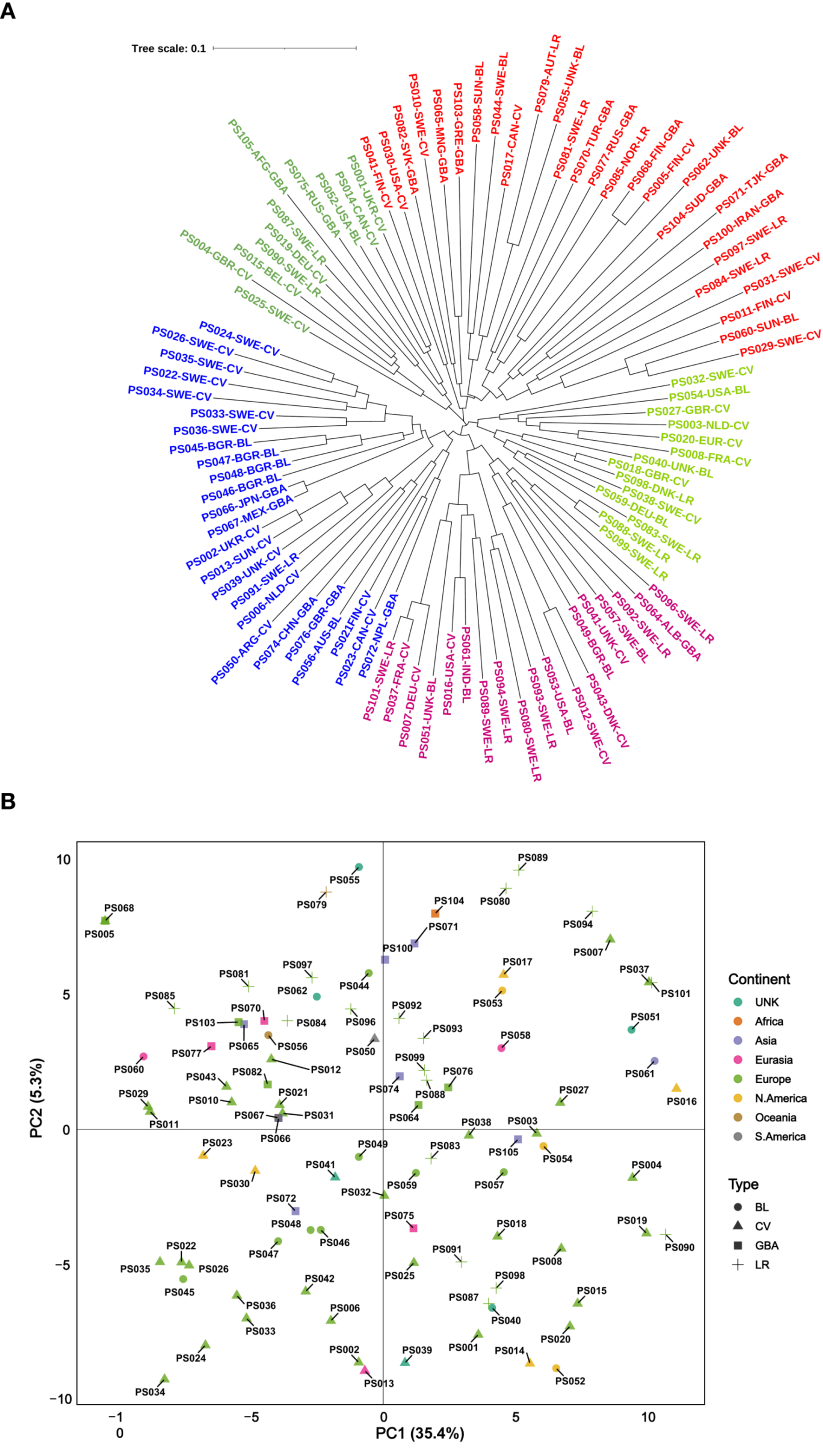
Genetic variation among the 100 accessions based on **(A)** neighbor-joining-based clustering and **(B)** principal component analysis (PCA) with the first two PCs (PC1 and PC2). Accessions are distinguished by color according to continental origin and by symbols representing material type. BL, breeding line; CV, cultivar; LR, landraces; GBA, genebank accessions; UNK, unknown.

The 100 accessions included in the current study were selected from the previous study by Brhane and Hammenhag (2024) with the goal of capturing maximum genetic variation. Among the selected accessions, 44 were cultivars, 20 landraces, 19 breeding lines, and 17 were genebank accessions with unknown or undefined material types. Most of the accessions originated from Europe (66) followed by North America (9) and Asia (8). Additional accessions were sourced from Eurasia (6), South America (2), Oceania (2) and Africa (1) while the origin of six accessions remained unknown. A neighbor-joining-based analysis was performed using the 2,573 high-quality SNP markers that passed the filtering criteria. The resulting dendrogram did not reveal any distinct or well-defined major clusters; instead, accessions were dispersed across several small and scattered clusters (**Figure 3A**). Although minor clusters were observed, the overall pattern showed limited association with either the geographic origin or the material type. Most accessions were randomly distributed across the dendrogram, with no clear separation based on these factors. Similarly, principal component analysis based on the same SNP set showed a broad spread of accessions, regardless of origin or classification. The first two principal components explained 35.4% and 5.3% of the total variation, respectively, (**Figure 3B**) and no clear clustering plot by continent or material type was evident.

### Protein and starch content analysis

**Figure 4:**
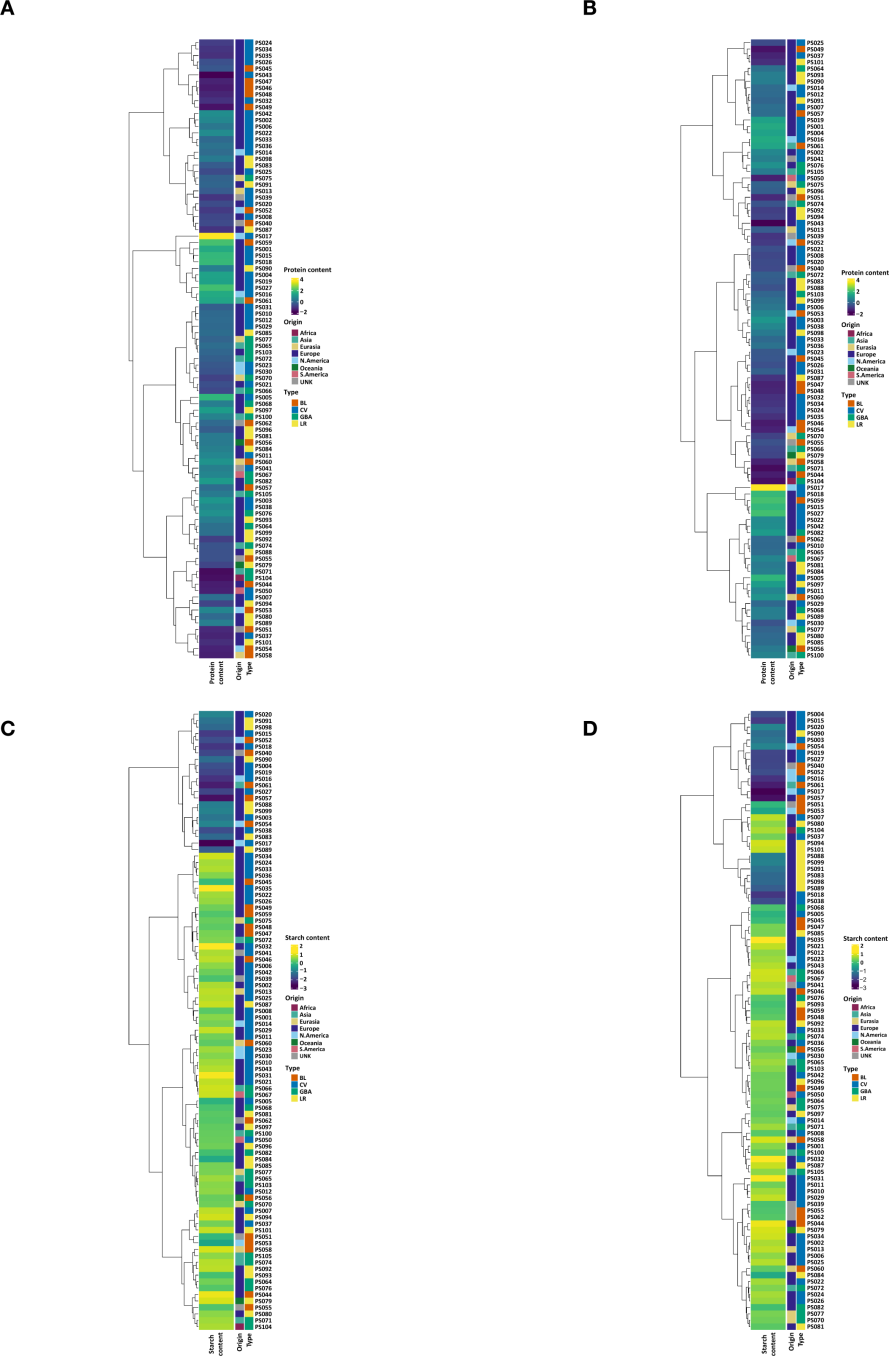
Dendrogram, heatmap and bar plots of the studied pea accessions. The first two figures comprised the dendrogram constructed using hierarchical clustering based on the first two principal components (PC1 and PC2) derived from all of the SNP markers **(A)** and subsets of SNP markers from storage protein genes **(B)** aligned with the protein content values of accessions. The last two figures encompassed the dendrogram constructed by hierarchical clustering with the first two PCs derived from all SNPs **(C)** and subsets of SNPs from starch enzymes **(D)** aligned with the starch content values of accessions. PRT, protein content; STR, starch content; UNK, Unknown; BL, breeding line; CV, cultivar, GBA, genebank accession; LR, landrace. The numbers along with the two letters PS represent the IDs of the accessions.

A high genetic variability was observed among the accessions for both starch and protein contents. The average protein content was 25.54% ranging from 19.47% to 37.69%, while starch content averages 40.09% with a range of 23.77% to 47.34%. Protein content was relatively consistent across cultivars, landraces and genebank accessions, with average values of 25.72%, 25.66% and 25.47%, respectively. Breeding lines exhibited a slightly lower mean protein content at 23.54%. To explore potential geographic trends, we compared average protein and starch contents among accessions grouped by continent of origin, noting that sample sizes varied and were in some cases small and non-comparable. Among the groups with comparable accession numbers, accessions from North America exhibited slightly higher average protein content than those from Eurasia and Asia (**Figure 4B**).

### Aligning protein and starch contents with SNPs in protein storage and starch enzyme genes

Dendrograms of the accessions were constructed based on the first two principal components (PCs) derived from three different marker sets; all SNP markers (2573), SNPs associated with storage protein genes (818) and SNPs associated with starch enzyme genes (1550). These clustering patterns were compared against the corresponding protein and starch content of the accessions (**Figure 4**; **Supplementary Figure 1**). The result revealed distinct clustering patterns linked to the two traits. In particular, the PCA based on all SNP markers exhibited a clear separation of accessions into two clusters based on starch contents (**Figures 4C, D**). Although the accessions did not form a single major cluster based on protein content, several smaller but distinct groupings were observed (**Figures 4A, B**).

### Effect of targeted genes on protein and starch contents

To further explore the genetic basis underlying the observed variation in protein and starch contents, a two-stage regression analysis was conducted to identify specific SNP markers and associated genes with significant effects on these traits. The analysis identified genes with both positive and negative effect on the protein and starch content (**Tables 2**, **3**). In the first stage of the regression analysis, 226 and 436 SNP variants were significantly correlated with protein and starch content, respectively (P < 0.05). Among these, 131 SNPs were common to both traits, and mapped to 25 genes, suggesting possible pleiotropic effects. These significant SNPs were distributed across all seven pea chromosomes, indicating a polygenic basis for the variation in these compositional traits.

**Table 2:** List of genes associated with significant SNP markers, and their estimated weighted average effects on protein content in the studied pea accessions.

No.	Gene name	No of SNPs^a^	Weighted avg. effect and SD^b^	No.	Gene name	No of SNPs^a^	Weighted avg. effect and SD^b^	No.	Gene name	No of SNPs^a^	Weighted avg. effect and SD^b^
1	provicilin-like_4	23	0.9 ± 2.6	12	legumin_A-like_3	7	2.4 ± 0.8	23	α-amylase	2	2.4 ± 0.04
2	conglutin_alpha 2-like	20	2.5 ± 0.6	13	legumin_A2-like	6	0.02 ± 3	24	β-amylase	2	3.5 ± 0.0
3	Provicilin-like_3	18	0.9 ± 2.5	14	provicilin-like_5	6	-0.2 ± 3	25	albumin_1 _A	1	2.4 ± 0.0
4	SSII	18	-0.3 ± 2	15	GBSSIb	5	-1.8 ± 0.0	26	albumin-1_D	1	-3 ± 0.0
5	convicilin_1	17	-1.8 ± 1.2	16	albumin-1_D-like	4	-1.1 ± 2	27	albumin-1-like_2	2	1.9 ± 0.3
6	convicilin_2	15	-2.4 ± 0.6	17	albumin-1_E	4	-3.1 ± 0.8	28	albumin-2	1	2 ± 0.0
7	AGPase_S2	14	1.5 ± 1.3	18	PGDH	4	3.2 ± 0.4	29	ISA3	1	-3.9 ± 0.0
8	SuSy1	13	-0.2 ± 1.7	19	albumin_1 _A_like	3	-3.3 ± 0.2	30	legumin_J_2	1	3.6 ± 0.0
9	SBEII	11	-3.2 ± 0.03	20	Provicilin	3	0.4 ± 1.8	31	SuSy3	1	3.8 ± 0.0
10	SBEI	10	0.2 ± 2	21	albumin-1-like_2	2	1.9 ± 0.3	32	vicilin-like_2	1	3.0 ± 0.0
11	albumin-1-like_1	9	-1.2 ± 1.8	22	Rb	2	3.04 ± 0.0				

**^a^**The number of significant SNP markers identified in each gene.

**^b^**The estimated weighted average effect and standard deviation of significant SNPs in a gene.

SD, standard deviation.

**Table 3:** List genes associated with significant SNP markers, and their estimated weighted average effect on starch contents in the studied pea accessions.

No.	Gene name	No of SNPs^a^	Weighted avg. effect and SD^b^	No.	Gene name	No of SNPs^a^	Weighted avg. effect and SD^b^	No.	Gene name	No of SNPs^a^	Weighted avg. effect and SD^b^
1	provicilin-like_4	47	-0.1 ± 5.7	13	provicilin-like_5	13	0.15 ± 4.9	25	SBEII	3	0.2 ± 3.7
2	SuSy2	39	-0.4 ± 3.7	14	legumin_A-like_3	11	-3.9 ± 0.9	26	albumin-1-like_2	2	2.2 ± 0.0
3	SSII	32	3.2 ± 1.1	15	legumin_B-like	11	-4.5 ± 0.9	27	PGMP	2	0.5 ± 0.0
4	SuSy1	31	-0.1 ± 3.4	16	Provicilin	11	-2.1 ± 3.4	28	albumin-2	1	-2.6 ± 0.0
5	conglutin_alpha 2-like	26	-2.8 ± 2.4	17	SuSy3	11	-1.8 ± 2.7	29	albumin-2-like_4	1	-2.9 ± 0.0
6	SBEI	25	-2.3 ± 4.2	18	albumin-1_D	10	-4.3 ± 1.5	30	GBSSIa	1	2.8 ± 0.0
7	albumin-1_D-like	21	-3.66 ± 3.4	19	PGDH	10	-4.8 ± 1.9	31	α -amylase	1	-5.2 ± 0.0
8	albumin-1_C	18	-7.5 ± 1.9	20	provicilin-like_1	9	-3.7 ± 0.3	32	legumin_A-like_1	1	-3.3 ± 0.0
9	GBSSIb	18	2.7 ± 2.2	21	AGPase_S2	8	-3.1 ± 0.3	33	provicilin-like_2	1	-7.2 ± 0.0
10	provicilin-like_3	18	2.4 ± 5.4	22	albumin-1_E	8	-4.7 ± 1.7	34	rb	1	-3.5 ± 0.0
11	albumin-1_A	16	-5.4 ± 1.8	23	legumin_A2-like	7	4.8 ± 4.7	35	Vicilin_chain_C	1	-7.0 ± 0.0
12	albumin-1-like_1	14	1.3 ± 4.9	24	albumin_1 _A_like	6	-2.3 ± 4.5	36	vicilin_like_1	1	3.8 ± 0.0

**^a^**The number of significant SNP markers identified in each gene.

**^b^** The estimated weighted average effect and standard deviation of significant SNPs in a gene.

SD, standard deviation.

A higher number of significant SNP markers were identified in the *legumin* and *provicilin* genes, located on chromosomes 3 and 5 respectively, which exhibited strong positive effect on the protein content of the studied pea accessions (**Supplementary Table 3**). Mapping of the SNPs to their corresponding genes revealed 32 genes affecting protein content, each linked to at least one significant SNP detected in the first-stage analysis. Among these, *provicilin-like_4* harbored the highest number of significant SNPs (23), whereas eight genes were represented by only a single significant SNP (**Table 2**). Overall, genes represented by one or two SNPs tended to exhibit higher average effect on protein content, particularly *SuSy3*, *legumin_J_2* and *β-amylase*, with mean effects of 3.8, 3.6 and 3.5, respectively. Notably, *conglutin_alpha 2-like*, which included 20 significant SNP markers, also showed a relatively high weighted average effect (2.5). In contrast, SNPs associated with *ISA3*, *albumin-1_E* and *albumin_1 _A_like* exhibited the strongest negative effects on protein content, with average estimated values of -3.9, -3.1 and -3.3, respectively (**Table 2**).

Functional annotation using SnpEff classified the majority of significant SNP markers associated with protein content as modifiers (132), followed by those with low (64) and moderate (30) predicted effects. Moderate and low impact markers expressed major or minor changes in amino acid sequences, out of storage protein genes, convicilin 1 showed 12 modifications with 7 major amino acid changes followed by convicilin-2 (8) and conglutin alpha 2-like (12) with 3 major changes in each. Among starch enzymes, SBEII and SSII genes showed major change in amino acid sequences with 5 and 4 moderate effect markers with total of 9 and 6 modified amino acids, respectively. However, no SNPs were classified as having a high impact (**Supplementary Table 4**). Regarding starch content, a total of 436 significant SNP markers were mapped to 36 genes (**Table 3**). Among these, *provicilin-like_4* contained the highest number of significant SNPs (47) associated with starch content. However, *starch synthase II* (*SSII*) exhibited a higher weighted gene effect, aggregated from 32 SNPs. Similarly, *granule-bound starch synthase Ib* (*GBSSIb*) showed a notable weighted average effect based on 18 SNPs. In contrast, genes such as *albumin-1_C* and *albumin-1_A* demonstrated strong negative effects on starch content, with average effects of -8.1 and -6.6, respectively, aggregated from 18 and 16 SNPs (**Table 3**).

The functional annotation tool classified the majority of significant SNP markers associated with starch content as modifiers (297) followed by those with low (91) and moderate (47) effects. Additionally, one SNP marker, located in the *provicilin-like_5* gene on chromosome 5 was annotated as having a high but negative effect on starch content (**Supplementary Table 5**). Several moderate-effect SNPs caused amino acid substitutions in known storage protein genes. For example, a histidine to aspartic acid substitution in *conglutin_alpha 2-like* (Chr3:169385527) was among the most strongly associated with increased protein content (effect = 3.64, p = 0.007). Multiple substitutions were also identified in *convicilin_1* and *convicilin_2*, including Cys25Ser and Gly376Arg, which were associated with reduced protein levels (**Supplementary Table 4**).

### Seed morphology traits

The studied pea accessions were further characterized for seed size, shape and seed coat color to assess potential associations with protein and starch content (**Supplementary Table 1**). Notably, the wrinkled seed phenotype exhibited a highly significant (p < 0.001) negative correlation with starch content (r = -0.71, **Table 4**). A strong and statistically significant positive correlation was observed between seed shape and average seed size (r = 0.62, *p* < 0.001), as well as between seed wrinkling and average seed size (r = 0.64, *p* < 0.001). A moderate negative correlation was observed between starch content and seed shape (r = -0.53, *p* < 0.001). Seed coat coloration, categorized as either patterned or uniform, had a significant (p < 0.05) but negative correlation with starch content.

**Table 4:** Correlation coefficients between seed morphology traits and protein- and starch content in studied pea accessions.

	Protein content	Starch content	Seed shape	Seed wrinkling	Average seed size	Seed color	Patterned/uniform seed color
Protein content	1	-0.5119^***^	0.2654^**^	0.2528^*^	0.0978^NS^	0.3226^NS^	0.0955^NS^
Starch content	-0.5119^***^	1	-0.5294^***^	-0.7065^***^	-0.3822^***^	0.4665^**^	-0.2074^*^
Seed shape	0.2654^***^	-0.5294^***^	1	0.7271^***^	0.6165^***^	0.4079^*^	-0.1496^NS^
Seed wrinkling	0.2528^*^	-0.7065^***^	0.7271^***^	1	0.6368^***^	0.4162^*^	0.1457^NS^
Average seed size	0.0978^NS^	-0.3822^***^	0.6165^***^	0.6368	1	0.3938^*^	0.1281^NS^
Seed color	0.3226^NS^	0.4665^**^	0.4079^*^	0.4162	0.3938	1	0.8595^***^
Patterned/uniform seed color	0.0955^NS^	-0.2074^*^	-0.1496^NS^	0.1457^NS^	0.1281^NS^	0.8595^***^	1

Level of significance: *** < 0.001; ** < 0.01; * < 0.05; NS, non-significant.

**Figure 5:**
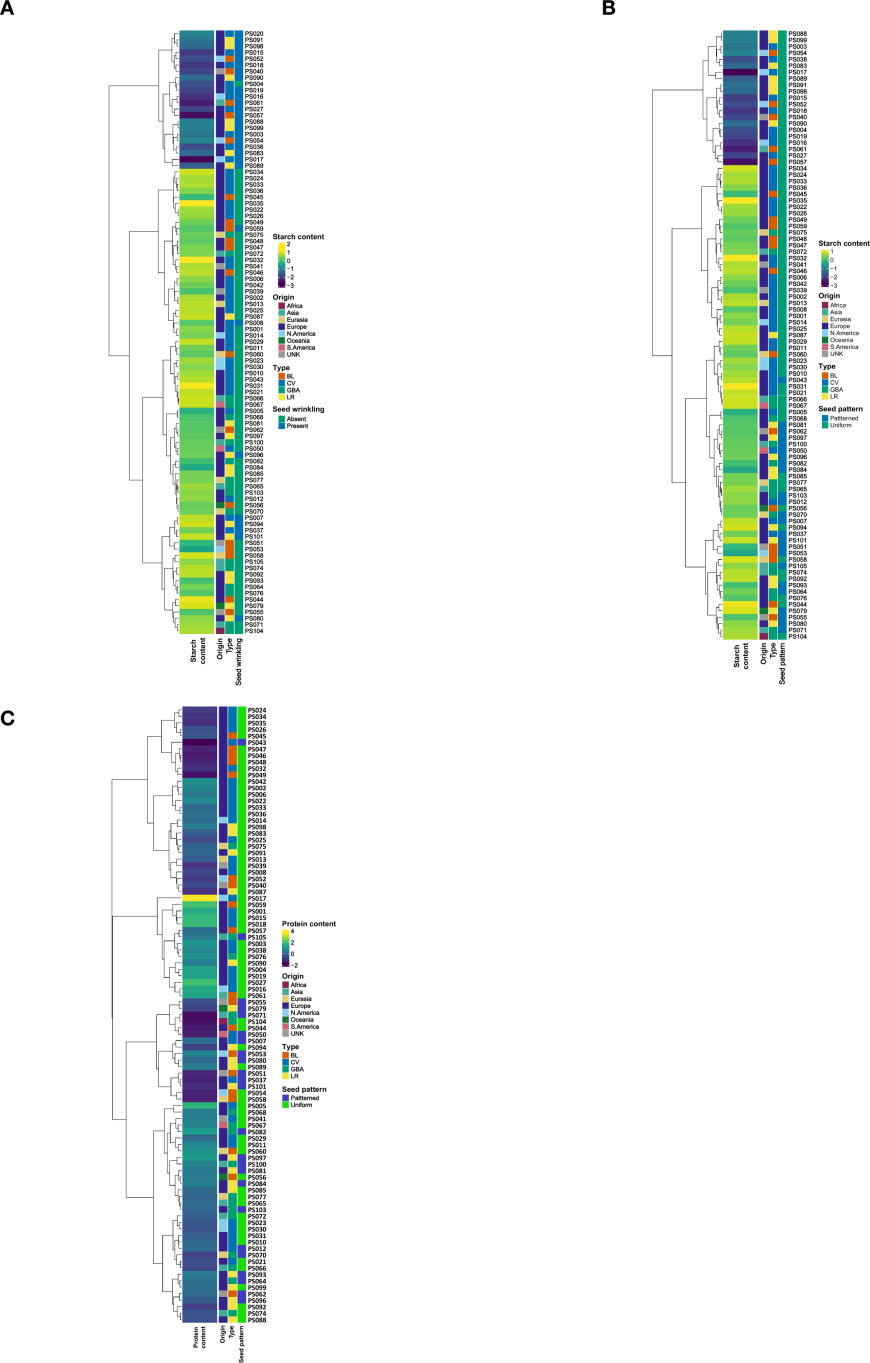
Aligning SNP marker profiles, protein and starch contents and seed morphology parameters of pea accessions using dendrogram, heatmap and bar plots. The figures comprised a dendrogram constructed using hierarchical clustering based on the first two principal components (PC1 and PC2) derived from all SNP markers. **(A)** Starch content vs seed wrinkling; **(B)** Starch content vs seed pattern; **(C)** Protein content vs seed pattern.

To further explore these relationships, the accessions were aligned with the PCs derived from the complete SNP dataset, along with their respective protein and starch content values and seed morphology traits. Of the 19 pea accessions clustered together in the low-starch content group, 18 exhibited the wrinkled seed phenotype (**Figure 5A**). All 19 accessions within this cluster exhibited a uniform seed color (**Figure 5B**). Additionally, accessions with relatively lower protein content were predominantly characterized by a patterned seed coat (**Figure 5C**).

## Discussion

Based on a comprehensive literature review, we identified 64 genomic regions for targeted sequencing, corresponding to 55 genes: 34 encoding storage proteins and 21 encoding enzymes involved in the starch biosynthesis pathway. This targeted-genes sequencing led us to the identification of 2,573 high-quality SNP markers from an initial set of 8,793 genetic variants, distributed across the seven pea chromosomes. The resulting SNP dataset was used in PCA and neighbor-joining clustering analysis, revealing a highly complex genetic structure among accessions, consistent with previous reports of weak clustering among globally collected pea germplasm (Solberg et al., 2015; Alemu et al., 2022; Brhane and Hammenhag, 2024). Our findings build on earlier studies that have effectively targeted specific genomic regions for population and trait-level analysis, providing valuable insights for investigating genetic variability and trait heritability in pea (Thévenot et al., 2005; Calenge et al., 2006; Jha et al., 2015; Carpenter et al., 2017).

The SNP markers derived from the sequencing of storage protein and starch enzyme-targeted genes effectively classified the 100 studied pea accessions based on their protein and starch content profile. This demonstrates the utility of gene-targeted sequencing for identifying genetic variants associated with starch and protein metabolism. Consistent with previous studies (Jha et al., 2015) the SNP variants identified in our study hold significant potential as molecular markers for genomic and marker-assisted selection, facilitating the improvement of starch and protein content in pea breeding programs. The clustering patterns observed in **Figure 4B** (based on SNPs in storage protein genes) and 4D (based on SNPs in starch enzyme genes) suggest that protein and starch content are influenced by distinct genetic factors. If the same accessions had clustered similarly in both figures, this would have indicated a shared or overlapping genetic basis. However, the observed differences in clustering suggest that the genetic determinants of protein and starch content are largely independent. This distinction is biologically plausible and aligns with findings in model plant systems, where storage protein genes are involved in nitrogen assimilation and protein synthesis, while starch enzyme genes function in carbohydrate metabolism and energy storage (Rolletschek et al., 2005). These pathways are regulated by different metabolic signals and controlled by largely separate transcriptional networks (Tayade et al., 2019a). The implication for breeding is that improving one trait may not necessarily affect the other, and targeted strategies are required if simultaneous improvement of both seed protein and starch content is a specific breeding objective.

Furthermore, the weak clustering by geographic origin or material type observed across analyses suggests that the genes studied here may be under similar selection pressures or widely conserved across germplasm. This pattern could reflect historical breeding practices or gene flow, focused on traits unrelated to storage protein or starch. Notably, accessions classified as modern cultivars did not show a clear tendency to cluster toward higher protein content, indicating that recent breeding efforts may not have prioritized protein improvement. However, some of these cultivars are vining or fresh-market types (e.g., garden peas harvested at the green immature stage) which are likely selected for traits such as sweetness or tenderness, where higher sugar content, rather than protein, has been the primary breeding target. Therefore, the results could be different if only dry pea cultivars were included. Nonetheless, this highlights the potential value of underutilized or less-advanced accessions as reservoirs of favorable alleles for protein content. Introgression of such alleles into elite backgrounds could offer a strategic route for enhancing the nutritional quality of future pea cultivars. Genomic analyses in several legume crops have identified important heritable genetic polymorphisms in genes encoding seed storage proteins (Warsame et al., 2018; Verma and Bhatia, 2019; Jha et al., 2022). These proteins significantly contribute to seed nutritional quality and functional properties, such as gel formation, making them important for different food applications. In our study, chromosomes 5 and 3 were identified as hotspots for highly significant SNPs in the legumin and provicilin genes. Several studies have reported these chromosomes as source of quantitative trait loci for protein content in pea (Irzykowska and Wolko, 2004; Burstin et al., 2007; Krajewski et al., 2012; Gali et al., 2019). These findings reinforce the value of these genomic regions as promising targets for marker-assisted selection in efforts to enhance protein content and functionality in future pea cultivars.

In this study, several moderate- and high-effect SNPs were detected in both storage protein and starch biosynthesis genes. Although detailed protein structure modelling was beyond the scope of this work, some of the amino acid substitutions we observed are likely to influence key biochemical properties. For storage proteins such as vicilin, convicilin, legumin and conglutin-α2-like, substitutions that alter amino acid charge or polarity may affect protein solubility, aggregation behavior and thermal stability, traits known to determine processing performance and texture of pea ingredients (Grossmann, 2024). Similarly, missense variants identified in starch enzymes such as SSII, SBEII and GBSSI could influence chain-length distribution and branching patterns of amylopectin, thereby modulating starch gelatinisation and seed textural properties (Tayade et al., 2019a; Yu et al., 2021). A similar pattern has been reported in other crops such as rice and maize, where mutations or allelic variations in the SBEII gene lead to protein changes. Although the overall enzymatic function is impaired, specific alterations in protein–protein interactions within the starch synthesizing complex leads to altered amylopectin chain length in the endosperm, thereby affecting starch granule morphology and composition (Nishi et al., 2001; Liu et al., 2012). Taken together, while the functional impact of these variants requires future biochemical validation, their occurrence in key metabolic genes provides a plausible molecular basis for the observed variation in seed composition.

Globulins, a group of plant proteins soluble in saline solutions, are the most abundant storage proteins in legumes (Kawakatsu and Takaiwa, 2017; Heldt and Piechulla, 2021). In pea seeds, legumin and vicilin are the predominant globulins (Heldt and Piechulla, 2021; Shaghaghian et al., 2022). These storage proteins are encoded by multigene families comprising at least 40 genes that exhibit substantial genetic polymorphisms (Casey et al., 2001). While previous studies have identified regulatory genes as primary determinants of protein composition in pea (Barac et al., 2010), our findings indicate that polymorphisms within the storage protein genes themselves (e.g., legumin and provicilin) also contribute significantly to variation in protein content. Indeed, the SNP markers we identified in these genes not only showed strong associations with protein content but also effectively grouped accessions based on this trait. This suggests that variation within the structural genes themselves plays a direct role in shaping seed protein levels, beyond the influence of regulatory pathways. In addition, conglutin, previously identified as a major protein component in lupin (Hall et al., 2017; Keuleyan et al., 2023) also showed significant SNPs association with protein content in our study.

In contrast to the positive effects of polymorphisms in storage protein genes, the second starch branching enzyme (SBEII), which plays a significant role in starch biosynthesis (Jha et al., 2015), exhibited a significant negative effect on protein content of the accessions studied. SBEII catalyzes the formation of α-1,6-glycosidic bonds in amylopectin which is a major component of starch (Seo et al., 2002). Daba et al. (2025) pointed out that wrinkled peas with defective SBEII activity showed a higher prevalence of vicilin compared to round peas, highlighting protein composition changes due to altered starch biosynthesis.

This interplay between starch metabolism and protein accumulation reflects a broader physiological balance during seed development. The inverse relationship between starch and protein accumulation in legume seeds is driven by shared carbon and energy resources and by the temporal regulation of metabolic genes. In our study, variation in SBEII showed opposite effects on protein and starch contents, supporting the idea that changes in starch branching can redirect carbon flux toward nitrogenous compounds. Previous work has shown that wrinkled pea mutants defective in SBEII accumulate less starch and relatively more protein (Daba et al., 2025), highlighting the integrative nature of carbon–nitrogen partitioning. Comparative transcriptome analyses in other legumes have similarly indicated that starch biosynthetic genes are upregulated during late seed filling in high-starch species such as adzuki bean, whereas they are downregulated in high-protein legumes such as soybean (Yang et al., 2015). Such regulatory shifts reflect a developmental trade-off between carbohydrate and protein deposition, which can be modulated by allelic variation in key starch enzymes.

Understanding the regulatory coordination between starch and protein pathways in pea could open new avenues for breeding dual-purpose cultivars that optimize both nutritional and functional seed traits.

The significant correlations observed between seed morphology traits and seed composition suggest potential interrelationships (Burstin et al., 2007). The positive correlations between seed shape and seed size, as well as between seed wrinkling and seed size, may reflect underlying genetic or developmental linkages influencing these traits. For instance, our study confirmed the well-known strong negative correlation between seed wrinkling and starch content (**Table 4**). Similarly, various previous findings have shown that wrinkled seeds significantly reduce starch accumulation compared to smooth seeds due to alterations in starch biosynthesis pathways (Ratnayake et al., 2002; Wang et al., 2011; Yu et al., 2021). Our result support these earlier findings, highlighting that key enzymes in starch biosynthesis collectively influence seed composition and food quality traits. Previous studies have also linked allelic variation in these starch biosynthesis genes to physicochemical properties such as amylose content and gel consistency (Han et al., 2019; Tayade et al., 2019a; Yu et al., 2021). In line with this, our analysis identified significant SNP associations for starch content on chromosomes 3, 5, and 7, supporting earlier reports of these loci as key regions for starch-related traits in pea (Gali et al., 2019). Notably, the SNP markers we identified on these chromosomes were significantly associated to starch contents across the analyzed pea accessions (**Supplementary Table 4**). Taking together, our findings highlight the potential of combining genetic insights from both starch and protein pathways to inform trait-targeted breeding strategies. By integrating genomic variation from underutilized germplasm with functional gene markers, future pea breeding can be guided toward developing cultivars that more efficiently meet nutritional, industrial, and market-specific demands.

## Conclusion

This study provides a comprehensive genetic analysis of key storage protein and starch biosynthesis genes in pea. SNP-based PCA and clustering analysis revealed distinct patterns among accessions closely aligned with differences in seed protein and starch content. These patterns support the functional relevance of the targeted genes in shaping these nutritional traits. It also highlight the potential of gene-targeted sequencing in identifying genetic variants underlying the target traits. Gene-to-phenotype association analysis identified significant polymorphisms in genes such as legumin, provicilin and SBEII, highlighting their pivotal role in determining protein and starch levels. Additionally, the strong negative correlation between seed wrinkling and starch content suggests that genetic variation in starch biosynthesis genes may also influence seed coat texture.

Together, these findings provide valuable genetic insights and molecular markers for use in breeding programs aimed at improving nutritional quality and processing characteristics in pea. Future research should focus on the functional validation of key candidate genes and integrate them into genomic or marker-assisted selection strategies to accelerate the development of improved pea cultivars for sustainable food production and industrial applications.

## Data Availability

The datasets presented in this study can be found in online repositories. The names of the repository/repositories and accession number(s) can be found in the article/**Supplementary Material**.
