# Supplementary material for: Genetic variation in storage protein and starch biosynthesis genes reveals key associations with seed composition in pea (Pisum sativum)
Source: Front Plant Sci. 2025 Dec 5;16:1679498. doi: 10.3389/fpls.2025.1679498 (PMC12716155; doi:10.3389/fpls.2025.1679498)
Supplement: Supplementary file 6 [file DataSheet1.pdf]

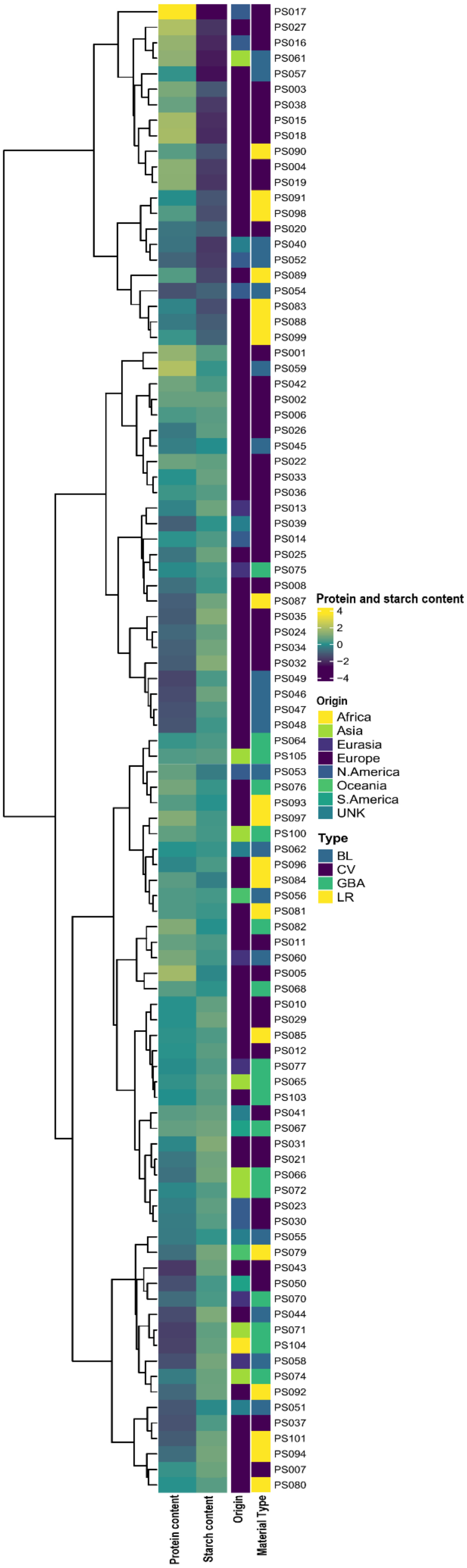

Supplementary figure 1. Dendrogram, heatmap and bar plots of the studied pea accessions. The figure encompassed the dendrogram constructed using hierarchical clustering based on the first two principal components (PC1 and PC2) derived from all the SNP markers, heat maps resulting from scaled values of protein and starch contents and barplots for origin of continent and material types.
